# Socioeconomic context of the community and chronic child malnutrition in Colombia

**DOI:** 10.11606/S1518-8787.2018052000394

**Published:** 2018-07-13

**Authors:** Ana María Osorio, Gustavo Alfonso Romero, Harold Bonilla, Luis Fernando Aguado

**Affiliations:** I Pontificia Universidad Javeriana Seccional Cali. Departamento de Economia. Cali, Colombia; II Institución Universitaria Antonio José Camacho. Facultad de Ciencias Empresariales. Cali, Colombia; III Pontificia Universidad Javeriana. Maestria en Economía. Bogotá, Colombia

**Keywords:** Child, Preschool, Child Nutrition, Malnutrition, Epidemiology, Risk Factors, Socioeconomic Factors, Health Inequalities, Preescolar, Nutrición del Niño, Desnutrición, Epidemiología, Factores de Riesgo, Factores Socioeconómicos, Desigualdades en la Salud

## Abstract

**OBJECTIVE:**

To analyze the influence of the socioeconomic context of the community on chronic child malnutrition in Colombia.

**METHODS:**

We estimated multilevel logistic models using data from the National Demographic and Health Survey in Colombia in 2010. The final sample included 11,448 children under the age of five gathered in 3,528 communities. In addition, we used the Principal Component Analysis with polychoric correlations for the construction of composed indicators of wealth, autonomy of the woman, and the use and access to the health system.

**RESULTS:**

The average level of community wealth was significantly and independently associated with chronic malnutrition in early childhood, more than the socioeconomic status of the household itself. At the individual and household level, the probability of chronic malnutrition was higher for children from mothers with low levels of autonomy and use and access to the health system, mothers who had their first child in adolescence, and mothers who live in homes in the lowest wealth quintiles. In contrast, children from mothers with a body mass index > 25 and with at least secondary education (versus no education) were less likely to suffer from chronic malnutrition.

**CONCLUSIONS:**

Research, programs, and interventions need to take into account the physical, economic, and social context of communities to contribute with the improvement of the nutritional status of early childhood in Colombia.

## RESUMEN

**OBJETIVO:**

Analizar la influencia del contexto socioeconómico de la comunidad sobre la desnutrición infantil crónica en Colombia.

**MÉTODOS:**

Utilizando datos de la Encuesta Nacional de Demografía y Salud en Colombia en 2010, se estimaron modelos logísticos multinivel. La muestra final incluyó 11.448 niños menores de cinco años anidados en 3.528 comunidades. Además, se utilizó el Análisis de Componentes Principales usando correlaciones policóricas para la construcción de indicadores compuestos de riqueza, autonomía de la mujer y el uso y acceso al sistema de salud.

**RESULTADOS:**

El nivel de riqueza promedio de la comunidad resultó significativa e independientemente asociado con la desnutrición crónica en la primera infancia, por encima y más allá del propio estatus socioeconómico del hogar. A nivel individual y del hogar, la probabilidad de desnutrición crónica fue mayor para niños de madres con bajos niveles de autonomía y uso y acceso al sistema de salud, que han tenido su primer hijo en la adolescencia y que viven en hogares en los quintiles más bajos de riqueza. En contraste, niños de madres con un índice de masa corporal > 25 y con al menos educación secundaria (*versus* sin educación) tuvieron menor probabilidad de padecer desnutrición crónica.

**CONCLUSIONES:**

Investigaciones, programas e intervenciones que tengan en cuenta el contexto físico, económico y social de las comunidades son necesarias para contribuir a mejorar el estado nutricional de la primera infancia en Colombia.

## INTRODUCTION

Chronic malnutrition (short height-for-age) is a key indicator to measure child well-being, as well as the progress of a country. It is an important economic variable that reflects health conditions, accumulation of human capital, and poverty^1^. Children with growth retardation are more likely to die during the first five years of life, to get sick, and to perform poorly at school. On the other hand, they have fewer economic opportunities in adulthood and are more prone to obesity and chronic diseases^2^.

Approximately 156 million children under the age of five in the world suffered from growth retardation in 2015, which is equivalent to 23.2% of the minors in this age group. This number was 12.7% in Colombia in 2010, which is slightly higher than the average for the region of Latin America and the Caribbean (10.0% – 2015), lower than for countries such as Peru (18.4% – 2012) and Ecuador (25.2% – 2012), and well above Chile (1.8% – 2014) and Brazil (7.0% – 2006)^a^.

Although the average levels of chronic malnutrition has decreased in the last decades in the country, great territorial inequalities still persist. The percentage of children with chronic malnutrition varies by state (first administrative division), between 3.8% (San Andrés and Providencia) and 34.7% (Vaupés)^b^. More recent figures confirm the ongoing challenge faced by Colombia in reducing social inequalities based on place of residence. There were 170 probable cases of deaths from malnutrition and associated to it in the country between January and July, 2016; almost 40% of them are concentrated in three states: Guajira, Vichada, and Chocó^c^.

The context in which the children live affects their health, regardless of the characteristics of the children, their mother, and their household^3–5^. Communities share physical, social, and economic attributes that may be fundamental to improve the health condition of individuals^6^. The sanitary conditions^1^, education^7^, social networks^8^, and socioeconomic status^9^ of communities are factors that are associated with the nutritional status of children.

The determinants of child malnutrition in Colombia have been analyzed mainly from an individual perspective. Few studies^10^ ^,^ ^11^ have explored the independent role that communities may have on nutritional status of children^d^.

The objective of this study was to analyze the influence of the socioeconomic context of the community on chronic child malnutrition in Colombia. The analysis is needed for the design of policies and the implementation of more effective programs to reduce social and health inequities in Colombia.

## METHODS

The data used in this study come from the National Demographic and Health Survey (ENDS) 2010 for Colombia. This is the fifth research conducted in the country by the *Profamília* ^e^ since 1990 within the framework of the Demographic and Health Surveys (DHS)^f^ program. The research is cross-sectional, with national coverage and representative at the urban and rural level, including six regions, 16 subregions, and the 33 states of the country included the capital: Bogotá D.C. The sample is probabilistic, clustered, stratified, and multistage. The selection at each stage was strictly random, and 51,447 households in 258 municipalities were effectively interviewed (91.6% response rate). Data were collected from 53,521 women (94.1% response rate) of childbearing age (13–49 years) and from all children under the age of five, including weight and height. Anthropometric measures were taken by nutritionists following a standardized manual. Weight was measured with an electronic scale (model 872; Seca) with a capacity of 200 kg and an accuracy of 50 g (weights from zero up to 50 kg) and 100 g (weights from 50 kg up to 200 kg), with tare function (mother or baby function). Persons wore light clothing without shoes and babies had no diaper. In the case of height, portable wooden measuring devices were used. One brand was Diseños Flores S.R. Ltda. (Peru) with a maximum capacity of two meters and an accuracy of one millimeter, and three other measuring devices were Weigh Measure, LLC, with a maximum capacity of 1.97 m and accuracy of one millimeter, all with functionality for adults and children. Children older than two years were measured in the standing position, and children under the age of two years were measured in the lying position.

Within municipalities, households were grouped into segments or contiguous groups with an average size of 10 dwellings. These groups or Primary Sampling Units (PSU) were used in this work as community proxy.

**Figure 1:**
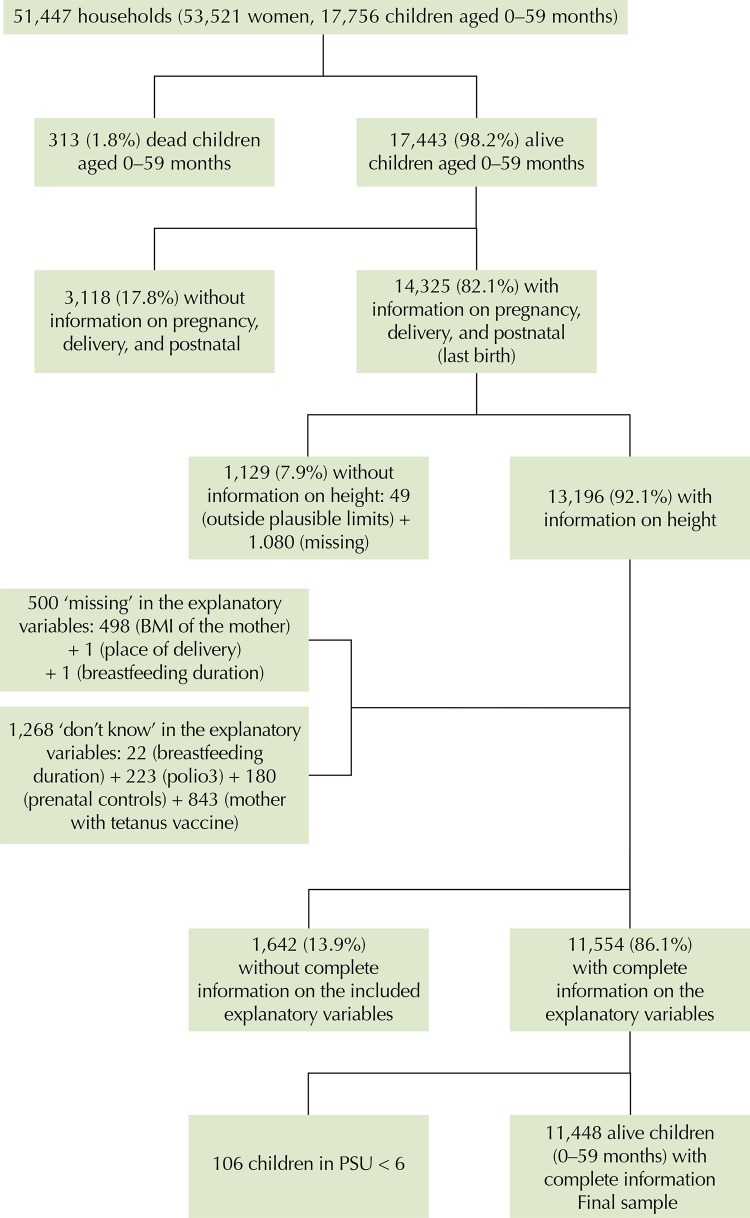
Sample selection flow, National Demographic and Health Survey. Colombia, 2010.

BMI: body mass index; PSU: primary sampling unit

Figure 1 presents the sample selection process. The ENDS 2010 included 17,756 children under the age of five, of which 17,443 were alive at the time of the interview. As part of the research project, information on prenatal care and delivery and postpartum conditions was collected only for the last child born alive (n = 14,325). The percentage of children in the survey without anthropometric measurement was 7.9%, which reduced the sample to 13,196 children. The “do not know” answers and “missing” data of the explanatory variables included in the analysis were excluded from the sample without significant differences between these cases and those included in the final sample (n = 11,554). In addition, in order to better capture the role of the community context, the PSU smaller than six were excluded. The sample included 11,448 children between zero and 59 months who were alive at the time of the interview and for whom complete information was present, distributed in 3,528 communities.

The dependent variable was chronic malnutrition (yes; no). We considered a child suffering from chronic malnutrition or growth retardation when the Z-score for height-for-age was below -2 standard deviations from the reference median set by the latest growth standards of the World Health Organization (WHO), 2006.

**Figure 2:**
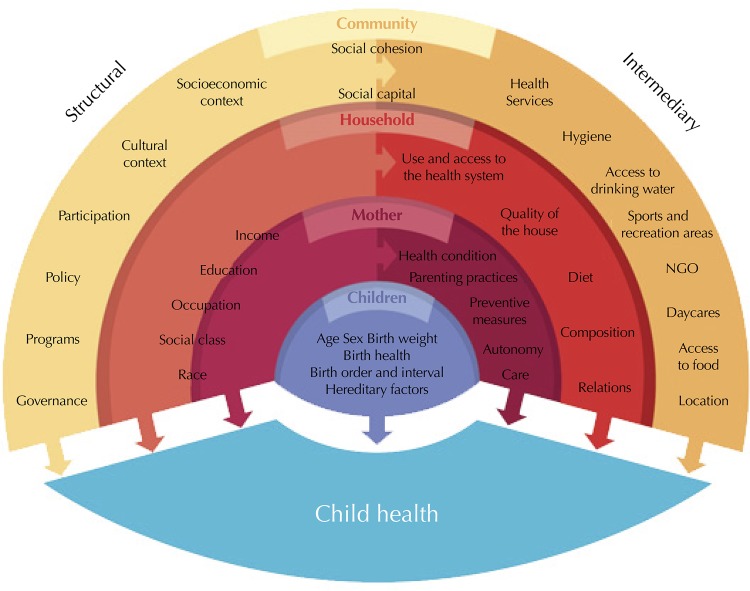
Conceptual framework for social determinants of child health.

Supported by the UNICEF^g^ conceptual framework for chronic malnutrition, the conceptual framework proposed by Mosley and Chen^12^, and the conceptual framework of the WHO Commission on Social Determinants of Health^13^, we constructed a conceptual framework that included structural and intermediary determinants of child health at different levels of analysis: children, mother, household, and community (Figure 2). Following this milestone and the literature review, we selected twenty variables available in the ENDS 2010 as potential explanatory variables. The variables were categorized and selected from the bivariate analysis (chi-square test p < 0.05).

The characteristics of the children considered were^h^: sex, age, breastfeeding duration, and interval from the previous birth. The characteristics of the mother were: the highest level of education achieved, age at first birth, Body Mass Index (BMI), autonomy in decision making, and use and access to the health system. The characteristics of the household were: number of children under the age of five and level of wealth (categorized into quintiles).

The characteristics of the community examined were: average level of wealth, education and autonomy of other women, hygiene conditions (access to drinking water and basic sanitation), and place of residence (urban or rural). The variables of this level were calculated from the aggregation of the data at the individual level considering the information of all the women included in the sample (n = 53,521). To avoid overlap between the two levels of analysis (level-1: children, mother, household, and level-2: community), variables were constructed using non-self-weighted averages. Finally, they were centered on the overall mean. The basic sanitation variables were highly correlated with the level of wealth (Pearson correlation coefficient *r* > 0.5), thus they were excluded from the final multilevel models.

The autonomy of the women in decision making was measured using a composite indicator that combined, in a single measure, information on whether the woman is alone or with someone who has the last word on: her own health, large household purchases, daily household shopping, visit to family members, what food to cook each day, study, and sexual intercourse. The indicator of use and access to the health system was a composite indicator that included: number of prenatal visits during pregnancy, whether the mother received the tetanus vaccine, whether the birth was performed by a physician or health professional, the place of birth, whether the child received the third dose of the polio vaccine, and whether the child had a vaccination card or not. The indicator of household wealth was a composite indicator that combined housing characteristics (flooring material, main material of walls, and connection to electric power) and the possession of durable consumer goods (television, radio, refrigerator, motorcycle, and car).

The composite indicators were constructed using Principal Component Analysis (PCA) with polychoric correlations using the “*polychoricpca*” command in Stata v13. Compared to traditional PCA, this method takes into account the categorical nature of the variables and allows obtaining a larger proportion of the explained variation^14^.

We used multilevel models to analyze individual and contextual factors associated with chronic child malnutrition. Multiple regression models assume that all individuals in the sample are independent, ignoring the possibility of a hierarchical structure of the data (such as the Demographic and Health Surveys – DHS) and, therefore, the possibility that individuals belonging to the same group (cluster) can share characteristics that can influence their results at the individual level. From the statistical point of view, by not taking into account clustering, the standard deviations of the coefficients can be underestimated and consequently very narrow confidence intervals and very small p-values can be calculated, which in turn can lead to spurious results^15^.

Given the discrete nature of the dependent variable (whether the child suffers from chronic malnutrition or not), we estimated multilevel logistic models at two levels. At the first level, children, mothers, and households were grouped together^i^. In the second level, the primary sampling units (PSU) were used as community proxy.

If we denote *h*_ij_ as the health status of the individual (child) *i* in the group (community) *j*, *X*_ij_ as a set of level 1 explanatory variables and *Z*_j_ as a set of level 2 explanatory variables, for a binary response of *h*_ij_ , *E* (*h*_ij_ | *X*_ij_, *Z*_j_ , *u*_j_) = πij = *pr* (*h*_ij_ =1), the logit model of random intercept can be expressed as:

$$\log\left(\frac{\pi_{ij}}{1-\pi_{ij}}\right)=\beta_{0}+\sum_{k=1}^{p}\beta_{k}X_{kji}+\sum_{1=1}^{q}\beta_{1}Z_{ij}+u_{j}$$ (1)

Where u_j_ are level 2 residues, which are assumed to be independent and follow a normal distribution with zero mean and variance *σ* ^2^_u_: *u*_j_ ~ *N*(0, *σ* ^2^_u_).

In multilevel models, the relationship between the variation between groups and the variation between individuals can be expressed using the Variation Partition Coefficient (VPC), which measures the proportion of total variation that is explained by level 2 (community) characteristics. The VPC oscillates between zero and one, indicating that: zero, there is no difference between groups (all variability of the dependent variable is explained by characteristics of the first level), and one, there are no differences within the group. For example, a VPC of 0.08 would indicate that 8% of the variability in the dependent variable can be attributed to the characteristics of the group.

All descriptive statistics were weighted using the *svy* command in Stata v13. The models were estimated using the *meqrlogit* command in Stata v13.

## RESULTS

Approximately 12.3% of the children in the sample had chronic malnutrition. Their average age was 26.9 months. Approximately 2.3% were never breastfed and 36.2% had no siblings. Most had mothers who had secondary education (54.9%) and who had their first child when adolescents (51.9%). While 14.5% of the children lived in households in the highest wealth quintile, 31.3% lived in poor or very poor households. Children lived in communities where women had 8.9 years of education on average, 85.2% had access to drinking water, and 79.7% had access to basic sanitation. Most (72.3%) lived in urban areas (Table 1).

**Table 1:** Sample characteristics, National Demographic and Health Survey. Colombia, 2010. (n = 11,448)

Variable	Percentage	95%CI	p
Characteristics of the child			

Sex			0.016
Male	51.2	49.96–52.44	
Female	48.8		
Age (months) (mean = 26.9 - SD = 16.5)			< 0.001
0–11	22.9	21.91–23.96	
12–23	23.6	22.55–24.61	
24–35	20.9	19.94–21.94	
36–47	17.5	16.58–18.4	
48–59	15.1	14.28–16	
Breastfeeding duration (months) (mean = 12.6 - SD = 9.14)			< 0.001
Never breastfed	2.3	1.98–2.68	
≤ 6	30.3	29.18–31.49	
≥ 7	67.4	66.16–68.55	
Interval from previous birth (months)			< 0.001
First birth	36.2	36.92–39.42	
< 24	9.5	8.79–10.16	
≥ 24	52.4	51.14–53.63	

Characteristics of the mother			

Education level			< 0.001
No education	1.7	1.44–2.06	
Primary	24.4	23.18–25.68	
Secondary	54.9	53.5–56.21	
Higher	19.0	17.9–20.17	
Age at birth (years) (mean = 20.4 - SD = 4.6)			< 0.001
≤ 19	51.9	50.5–53.25	
20–29	42.9	41.58–44.21	
30–49	5.2	4.69–5839	
Body Mass Index (kg/m^2^) (mean = 25.06 - SD = 4.6)			0.045
< 18.5	4.3	3.85–4.81	
18.5 to 24.9	51.0	49.78–52.19	
≥ 25	44.7	43.5–45.92	
Level of autonomy in decision making			0.015
Low	39.3	38.06–40.54	
Average	25.3	24.27–26.41	
High	35.4	34.16–36.63	
Level of use and access to the health system			0.001
Low	14.0	13.09–14.9	
Average	26.3	25.21–27.5	
High	59.7	58.39–60.98	

Characteristics of the household			

Number of children < 5 years (mean = 1.4 - SD = 0.67)			
Wealth indicator (quintiles)			< 0.001
Very poor (q1)	12.2	11.27–13.08	
Poor (q2)	19.1	18.08–20.21	
Middle (q3)	26.1	24.89–27.27	
Rich (q4)	28.1	26.89–29.41	
Very rich (q5)	14.5	13.57–15.55	

Characteristics of the community			

Average level of wealth^a^ (mean = 0.046 - SD = 0.84)			
Education of the women (years) (mean = 8.9 - SD = 3.92)			
Average level of autonomy^a^ (mean = 0.023 - SD = 0.56)			
% of households with access to drinking water	85.2	84.13–86.29	
% of households with access to basic sanitation^b^	79.7	77.89–80.95	
Place of residence			< 0.001
Rural	27.67	25.85–29.57	
Urban	72.33	70.43–74.15	

^a^ Composite indicators centered on the overall mean.

^b^It includes sewage network and garbage collection.

Table 2 presents the results of the multilevel logistic models for chronic malnutrition in children under the age of five in Colombia. Three models were estimated sequentially. Model 1, or null, did not include explanatory variables and allowed us to count the influence of the context. Model 2 (level 1), included the characteristics of the child, mother, and household. Model 3 (level 1 + 2), included the characteristics of the community to model 2.

**Table 2:** Multilevel logistic models for chronic malnutrition in children under the age of five. National Demographic and Health Survey. Colombia, 2010. (n = 11,448)

Variable	Model 1		Model 2		Model 3	
		
	OR	95%CI	OR	95%CI	OR	95%CI
Characteristics of the child						

Sex (Ref.: Female)						
Male			1.227^a^	1.09–1.37	1.228^a^	1.10–1.38
Age (months) (Ref.: 0–11)						
12–23			1.820^a^	1.52–2.18	1.820^a^	1.52–2.18
24–35			1.749^a^	1.45–2.11	1.754^a^	1.45–2.12
36–47			1.443^a^	1.18–1.77	1.453^a^	1.18–1.78
48–59			1.258^b^	1.01–1.57	1.269^b^	1.01–1.59
Breastfeeding duration (months) (Ref.: ≤ 6)						
Never breastfed			1.516^b^	1.03–2.23	1.488^b^	1.01–2.18
≥ 7			1.266^b^	1.09–1.47	1.235^b^	1.07–1.43
Interval from previous birth (months) (Ref.: First birth)						
< 24			1.268^b^	1.03–1.56	1.283^b^	1.05–1.57
≥ 24			0.984	0.86–1.13	0.982	0.85–1.13

Characteristics of the mother						

Education level (Ref.: No education)						
Primary			0.734^b^	0.54–0.99	0.777	0.57–1.05
Secondary			0.508^a^	0.37–0.69	0.553^a^	0.40–0.76
Higher			0.533^a^	0.37–0.76	0.584^b^	0.40–0.84
Age at birth (years) (Ref.: 20–29)						
≤ 19			1.146^b^	1.01–1.30	1.141^b^	1.01–1.30
30–49			0.855	0.60–1.23	0.866	0.60–1.24
Body Mass Index (kg/m^2^) (Ref.: < 18.5)						
18.5–24.9			0.928	0.70–1.23	0.924	0.70–1.22
≥ 25			0.782^c^	0.59–1.04	0.785^c^	0.59–1.04
Level of autonomy in decision making (Ref.: High)						
Average			1.052	0.90–1.23	1.046	0.89–1.23
Low			1.174^b^	1.02–1.35	1.156^b^	1.01–1.33
Level of use and access to the health system (Ref.: High)						
Average			1.101	0.95–1.28	1.097	0.95–1.27
Low			1.474^a^	1.27–1.72	1.385^a^	1.19–1.62

Characteristics of the household						

Number of children < 5 years			1.211^a^	1.12–1.31	1.195^a^	1.10–1.30
Wealth indicator (Ref.: Very rich q5)						
Very poor (q1)			2.973^a^	2.26–3.90	2.217^a^	1.64–3.00
Poor (q2)			2.170^a^	1.66–2.83	1.886^a^	1.43–2.48
Middle (q3)			1.643^a^	1.26–2.14	1.502^b^	1.15–1.96
Rich (q4)			1.492^b^	1.14–1.95	1.448^b^	1.11–1.89

Characteristics of the community						

Level of wealth					0.806^a^	0.72–0.90
Education of the women					1.003	0.96–1.05
Autonomy of the women					0.94	0.84–1.05
Access to drinking water					1.086	0.88–1.34
Place of residence (Ref.: Rural)						
Urban					1.108	0.94–1.31

Random effects						

Community-level variation (Standard Error)	0.662^a^	0.499–0.825	0.285^a^	0.179–0.453	0.251^a^	0.15–0.42
Variance Partition Coefficient (VPC)^d^	0.167		0.079		0.071	

^a^ p < 0.001

^b^ p < 0.05

^c^ p < 0.1

^d^ It measures the proportion of variation that is explained by differences between groups (communities).

After controlling for individual and community characteristics (model 3), the probability of chronic malnutrition increased with the child’s age (however, the relationship was not linear), and it was higher for males, for children with very short birth intervals, for children who have never been breastfed or who were breastfed for more than seven months (compared to those breastfed for six months or less), and for children whose mothers had low levels of autonomy and use and access to the health system and who had their children as adolescents. In contrast, children of mothers with a BMI greater than 25 and with secondary or higher education were less likely to suffer from chronic malnutrition. The level of wealth was positively and significantly associated with the probability of chronic malnutrition. Chronic malnutrition odds were 2.2 times higher for a child living in a very poor household compared to a child in a household in the highest wealth quintile.

Children living in communities with higher average levels of wealth were less likely to suffer from chronic malnutrition. The autonomy, education of the other women in the community, and access to drinking water did not show statistical significance in the complete model. When these variables were added separately^j^, we observed an association with chronic child malnutrition.

The last rows in Table 2 show the random effects of the model. The variance was statistically significant in all three models, which indicates significant unobservable heterogeneity in the delay in infant growth among communities. Model 1 showed that 16.7% of the residual variation in the probability of a child suffering from chronic malnutrition was attributable to the characteristics of the community. The inclusion of the characteristics of the child, mother, and household (model 2) reduced VPC by almost half (7.9%). When taking into account community-level variables (model 3), it was reduced to 7.1%.

## DISCUSSION

This study, in addition to analyzing individual and household factors usually associated with chronic child malnutrition, explored the relative contribution of certain socioeconomic characteristics of the environment where the child lives, which may help us better understand the social determinants of this health outcome in Latin American countries such as Colombia. In addition, the inclusion of variables such as the level of wealth, education, and empowerment of the women at both levels of analysis allowed us to explore the possibility of positive externalities, that is, the possibility that mothers, and therefore the children, benefit from the physical, economic, and social capital of their communities.

The contextual analysis showed significant variation in chronic child malnutrition among communities in Colombia. The association between the average level of wealth and the probability of a child suffering from chronic malnutrition was the only statistically significant association. This variable may be capturing the effect of important determinants of chronic malnutrition at the community level, such as access to drinking water, sanitation services, and education. Our results show that, in addition to the importance of the socioeconomic background of the family, the socioeconomic context of the community can contribute independently to the health status of the children, coinciding with that found in Latin America, Africa, and Asia^3^ ^,^ ^4^ ^,^ ^16^ ^,^ ^17^. Richer communities tend to have better physical infrastructure and services and greater access to food in both quantity and quality. This reduces the likelihood of getting sick and thus improves the health status of children^6^.

For the level of autonomy of other women in the community, the lack of association may be due to the fact that the level of wealth of the community is mediating the influence of this characteristic. A previous study for Colombia^11^ has shown that women, especially the poorest ones, can benefit from the level of autonomy of other women in their environment. At the individual level, however, our findings show that the autonomy of the mother contributes positively to the decrease in the probability of chronic malnutrition, even after being controlled by the socioeconomic status of the household and the education of the mother. The autonomy of the mother, understood as the ability of the mother to make decisions that affect her or her family, is an important predictor of the nutritional status of children^18^. Mothers with autonomy over the care of their children can make better decisions about diet, hygiene, care for their children, and the distribution of resources within the household^19^. It is also related to the decision about their own care, such as the use of contraceptives and the interval of birth between children. Mothers with greater autonomy tend to have greater access to health services, as well as to seek them timely, despite the cost that this implies^20^. They also have a lower risk of depression and anxiety^21^ ^,^ ^22^.

Our results confirm the importance of the education of mothers for the health of children, found in previous studies for Colombia^10^ ^,^ ^11^ and developing countries^23^ ^,^ ^24^. Education level may influence the health of the children from the access to information or the ability to acquire new knowledge. A higher level of education can mean greater knowledge about good eating practices, better health practices, as well as prevention and care of possible diseases. Our findings suggest that there is no significant difference in the probability of chronic malnutrition among children of mothers without education and those with primary education. There seems to be evidence that it is the mother’s literacy rather than her education level that contributes to the nutritional status of the children. In fact, after the mother reaches a certain education level, the contribution of a higher level to the probability of chronic malnutrition is minimal (secondary education OR = 0.55; higher education OR = 0.58). It would be interesting to explore this hypothesis in future studies or with different measures for education, which would allow a better approximation to reading comprehension and appropriation of knowledge. In developing countries, such as most Latin American countries, characterized by high levels of poverty and inequality and where the quality of education, especially public education, is not very good, a high education level does not ensure a better understanding or knowledge^25^.

Children whose mothers are overweight or obese (BMI > 25) are less likely to suffer from chronic malnutrition compared to children of extremely thin mothers. This probably indicates their possibility of accessing a greater amount of food^26^ ^,^ ^27^, but not necessarily healthy diets.

Our results show a social gradient of the level of household wealth and corroborate the importance of the use and access to the health system for the nutritional status of children, which are relations widely supported by the empirical literature^4^ ^,^ ^5^ ^,^ ^16^ ^,^ ^28^ ^,^ ^29^. However, when we included the characteristics of the community, the influence of these two indicators is attenuated. This suggests the importance that the social and physical environment of the community and its services can have in mediating the characteristics of the family. While household wealth influences the ability to access goods and services that promote better health, the health system itself is an intermediate determinant. Structural determinants operate through it to shape inequities in health^13^ ^,^ ^28^.

One of the strengths of this study is the use of a highly reliable and internationally comparable database. However, it is important to take into account some limitations. Because this is a cross-sectional study, we cannot establish causal relations. In addition, much of the data included is based on self-reported information from the women interviewed, which can lead to measurement errors. On the other hand, after being controlled by individual and community characteristics, there is still an unexplained percentage of residual variation that can be attributed to unobserved community-level factors that may be more difficult to capture, such as group practices, customs, synergies by affinities and close environments, and human capital endowments. It could also be attributed to the non-inclusion of social environment factors such as violence, social cohesion, and standards, or physical attributes, such as presence of markets, Non-Governmental Organizations (NGO), public spaces for recreation and sport, which are not usually included in DHS. Chronic malnutrition, in addition to being an internationally used indicator to measure child health status, is considered a key economic variable^1^, as it reflects the structural conditions of poverty and has irreversible consequences on the development of children. However, there are other variables that account for child health status, such as weight and anemia, which were not taken into account in this study. The period of the research is also a limitation. However, as of the date of completion of this article, the microdata of the ENDS 2015 for Colombia were not available. What we expect is that as soon as the information is available it will be possible to continue the study, systematically studying the role of the community context on the nutritional status of the children.

The characteristics of the household act together with the attributes of the socioeconomic context of the community on the nutritional status of the children – measured by height-for-age –, which indicates the importance of including both contexts in research, programs, and policies in Colombia. The context is especially important because it can act not only on individual outcomes, but also on other determinants of child health. The generation of household income needs to be promoted at the same time as the physical, social, and community resources. In addition, the empowerment of mothers in decision making needs to be promoted as well as their literacy. We hope that this work will contribute to a better implementation of the main strategy of the government to promote early childhood development, called “From Zero to Always” (recently raised to State Policy, Law 1804 of 2016), and the prioritization of the closest environment of the children. Key policy instruments can be developed taking into account the socioeconomic environment of the community, which can help to better define at what level (individual or community) interventions and programs must be carried forward to improve child health.

## Footnotes

a UNICEF; WHO; World Bank. Joint child malnutrition estimates: levels and trends. Geneva; 2016 [cited 2018 Jan 14]. Available from: http://data.unicef.org/nutrition/malnutrition.html

b Profamilia. Encuesta Nacional de Demografía y Salud ENDS Colombia 2010: informe. Bogotá (CO); 2011 [cited 2018 Jan 14]. Available from: https://dhsprogram.com/pubs/pdf/FR246/FR246.pdf

c Boletín Epidemiológico. Bogotá: Instituto Nacional de Salud. 2016;(30):74. Available from: http://www.ins.gov.co/buscador-eventos/BoletinEpidemiologico/2016%20Boletin%20epidemiologico%20semana%2030.pdf

d The work of García et al. (2013) uses data from the National Demographic and Health Survey - ENDS 2005. This survey, unlike ENDS 2010, did not included the rural population of the region of Orinoquía and Amazonía. The gains in the representativeness of the sample in 2010 allow us to obtain more precise estimates of the indicators. Furthermore, they do not take into account variables related to the use and access to the health system or the autonomy of women, which have been demonstrated as being associated with chronic child malnutrition in the literature. On the other hand, the work of Osorio et al. (2013), is focused on the education of the women in the community as the only contextual variable.

e Profamilia. Encuesta Nacional de Demografía y Salud 2005: salud sexual y reproductiva en Colombia. Bogotá; 2006 [cited 2018 Feb 7]. Available from: http://profamilia.org.co/docs/ENDS%202005.pdf

f The DHS is part of a program founded in 1984 by the United States Agency for International Development (USAID), to collect representative data at the national level in developing countries on population, maternal health, fertility, family planning, gender, HIV / AIDS, malaria and nutrition. DHS is widely recognized as the most important source of information for the analysis of health inequalities in the developing world. In Colombia, the DHS 2010 was funded by the Ministry of Social Protection and USAID, with the technical support of ICF Macro.

g UNICEF. Lineamientos estratégicos para la erradicación de la desnutrición crónica infantil en América Latina y el Caribe Tacro (PA); 2008 [cited 2018 Jan 14]. Available from: http://www.unicef.org/lac/final_estrategia_nutricion(2).pdf

h Birth weight is an important predictor of chronic malnutrition; however, it was not taken into account in the final models because of the large amount of missing data (25% of the total sample) in the ENDS 2010.

i Since the sample includes approximately one child per mother and one mother per household, these are considered part of the same level.

j Results not shown here.
